# PHYSICAL ACTIVITY AND SEDENTARY BEHAVIORS: IMPACT ON SELF-RATED HEALTH AND PHYSICAL OUTCOMES IN CANCER SURVIVORS

**DOI:** 10.1093/geroni/igz038.1422

**Published:** 2019-11-08

**Authors:** Shirley M Bluethmann, Eileen Flores, Charles Matthews, Frank Perna

**Affiliations:** 1 The Pennsylvania State University College of Medicine, Hershey, Pennsylvania, United States; 2 Penn State College of Medicine, Hershey, Pennsylvania, United States; 3 National Cancer Institute, Rockville, Maryland, United States

## Abstract

Physical activity (PA) and avoidance of inactivity are recommended in cancer survivorship. But survivors are not meeting these recommendations. We used national data (NHANES) collected 2011-2014 (n=9620) to estimate associations of PA and TV viewing with 3 health outcomes: self-rated health, functional limitations and multimorbidity in older cancer survivors and adults without cancer. Greater PA was associated with reporting excellent health in survivors. Survivors that obtained 22.5+ MET-hours/week were 5.5 times more likely to report excellent health than those that did no exercise (OR=5.5, p<.001). We observed a decrease in likelihood of multimorbidity and functional limitations with increasing PA (both significant at p<.001). We noted survivors that abstained from watching TV were 3x more likely to report excellent health and between 60-80% less likely to report functional limitations and multimorbidity than TV watchers (p<001). Findings with non-cancer adults were similar. Survivors need PA and reduced TV to maximize health outcomes.

